# Psoriasis Is Associated With Elevated Gut IL-1α and Intestinal Microbiome Alterations

**DOI:** 10.3389/fimmu.2020.571319

**Published:** 2020-10-01

**Authors:** Sergey Yegorov, Dmitriy Babenko, Samat Kozhakhmetov, Lyudmila Akhmaltdinova, Irina Kadyrova, Ayaulym Nurgozhina, Madiyar Nurgaziyev, Sara V. Good, Gonzalo H. Hortelano, Bakytgul Yermekbayeva, Almagul Kushugulova

**Affiliations:** ^1^School of Sciences and Humanities, Nazarbayev University, Nur-Sultan, Kazakhstan; ^2^Faculty of Education and Humanities, Suleyman Demirel University, Almaty, Kazakhstan; ^3^Karaganda Medical University Research Centre, Karaganda, Kazakhstan; ^4^Art Science LLP Innovative Center, Nur-Sultan, Kazakhstan; ^5^Laboratory of Human Microbiome and Longevity, National Laboratory Astana, Nazarbayev University, Nur-Sultan, Kazakhstan; ^6^Department of Biology, The University of Winnipeg, Winnipeg, MB, Canada; ^7^“University Medical Center” Corporate Fund, Nur-Sultan, Kazakhstan

**Keywords:** cytokines, Kazakhstan, Central Asia, mucosal immunity, gut microbiome, intestinal inflammation, psoriasis, skin disorder

## Abstract

**Background:**

Psoriasis is a chronic inflammatory condition that predominantly affects the skin and is associated with extracutaneous disorders, such as inflammatory bowel disease and arthritis. Changes in gut immunology and microbiota are important drivers of proinflammatory disorders and could play a role in the pathogenesis of psoriasis. Therefore, we explored whether psoriasis in a Central Asian cohort is associated with alterations in select immunological markers and/or microbiota of the gut.

**Methods:**

We undertook a case-control study of stool samples collected from outpatients, aged 30–45 years, of a dermatology clinic in Kazakhstan presenting with plaque, guttate, or palmoplantar psoriasis (*n* = 20), and age-sex matched subjects without psoriasis (*n* = 20). Stool supernatant was subjected to multiplex ELISA to assess the concentration of 47 cytokines and immunoglobulins and to 16S rRNA gene sequencing to characterize microbial diversity in both psoriasis participants and controls.

**Results:**

The psoriasis group tended to have higher concentrations of most analytes in stool (29/47 = 61.7%) and gut IL-1α was significantly elevated (4.19-fold, *p* = 0.007) compared to controls. Levels of gut IL-1α in the psoriasis participants remained significantly unaltered up to 3 months after the first sampling (*p* = 0.430). Psoriasis was associated with alterations in gut *Firmicutes*, including elevated *Faecalibacterium* and decreased *Oscillibacter* and *Roseburia* abundance, but no association was observed between gut microbial diversity or *Firmicutes/Bacteroidetes* ratios and disease status.

**Conclusions:**

Psoriasis may be associated with gut inflammation and dysbiosis. Studies are warranted to explore the use of gut microbiome-focused therapies in the management of psoriasis in this under-studied population.

## Introduction

Psoriasis is a chronic autoimmune condition that predominantly affects the skin and manifests with variable severity (1). The clinical classification of psoriasis is based on the pattern and extent of cutaneous involvement. For example, the most common phenotype, psoriasis vulgaris, or plaque-type psoriasis, is distinguished by the presence of well-defined areas of erythematous and indurated plaques and infiltration of the epidermis and dermis by mononuclear cells (1). Frequently, psoriasis is associated with extracutaneous manifestations, such as psoriatic arthritis (PsA) or cardiovascular disease, indicating that systemic inflammatory processes likely underlie psoriatic disease (1, 2).

The causes of psoriasis are incompletely understood; genetic predisposition plays a major role (1), but other factors such as systemic inflammation and microbiota alterations have also been implicated in disease pathogenesis. Furthermore, the link between psoriasis and inflammatory bowel disease (3) intriguingly points at the gut as an important contributor to psoriasis development. In support of this, recent studies have reported major alterations in both gut microbial communities (4–7) and markers of immune response (6) in individuals with psoriasis.

In earlier work, we characterized the gut microbiome of adults with and without metabolic syndrome from Kazakhstan, a country in Central Asia (8). As part of our overarching objective to better understand the relationship between the mucosal and systemic correlates of chronic disease in this region (8, 9), here we expand on our earlier findings and focus on the gut microenvironment in individuals with psoriasis. We hypothesized that psoriasis in adult Central Asians is associated with gut inflammation and microbiome alterations. To test this hypothesis, we assessed levels of select cytokines and immunoglobulins and performed a metagenomic analysis of stool samples obtained from dermatology clinic outpatients in the capital city of Kazakhstan.

## Materials and Methods

### Study Setting and Participant Recruitment

Participants aged 30–45 years were recruited through an outpatient dermatology clinic at the Centre for Dermatology and STD prophylaxis in the capital city of Kazakhstan, Nur-Sultan (formerly Astana). The dermatological assessment of participants was done in accordance with the national clinical guidelines for psoriasis diagnosis and treatment of Kazakhstan.

Study exclusion criteria were: use of antibiotics within 3 months prior to the study, a diagnosis of psoriatic arthritis, presence of any other chronic condition of the skin or gastrointestinal tract and presence of any severe comorbidity, or pregnancy. Healthy controls were recruited from the local communities through community-wide advertisement of the study and matched by age, sex, and ethnicity. A fecal sample was collected at baseline from all participants, and subsequently from the psoriasis patients at 6 and 12 weeks after the first visit. Fecal samples from all study participants were frozen within 2 h of collection and kept frozen at −80°C until analysis.

This study was designed as an exploratory case-control study to supply pilot data for future studies in the same population; therefore, no formal sample size calculations were performed and the sample size was determined based on the available study budget. In total, 40 individuals were included in the study (*n* = 20 with psoriasis, *n* = 20 controls). All experimental assays were performed by research personnel blinded to the psoriasis status of participants. All research procedures were approved by the institutional review board of the UMC CF Academic Council. Written consent to participate was collected from all participants. We used the STROBE checklist when writing our report (10).

### Cytokine Measurements

Frozen stool samples were thawed, and approximately 4 mg of each sample were dissolved in 200 μl of phosphate buffered saline. The supernatant obtained by centrifugation at 16,000 *g* for 15 min was then analyzed using the Milliplex Map Human Magnetic Bead Panels for cytokines and chemokines (HCYTMAG-60K-PX41) and immunoglobulins (HGAMMAG-301K-06) according to the manufacturer’s protocol on a Bio-Plex 3D instrument (Bio-Rad). The ELISA assay details and analyte classification are given in Supplementary Table 1. To avoid the bias of inter-plate variation, paired samples were examined on the same ELISA plate.

### Microbiome Analysis

#### DNA extraction and sequencing

A subset of 21 stool samples (14 psoriasis and 7 controls) from the first study visit was available for microbiome analysis. Samples were thawed prior to DNA extraction using QIAamp DNA Mini Kit (Qiagen). The quality of extracted DNA was assessed using Qubit dsDNA HS Assay Kit (Thermo Fisher) on a Qubit 2.0 according to the manufacturer’s manual (Invitrogen, Life Technologies). Next-generation sequencing libraries were prepared with NEXTflex 16S V1-V3 Amplicon-Seq Kit (PerkinElmer), and library quality assessed using the Qubit 2.0 system. Amplicons (96 samples per lane) were sequenced using the MiSeq platform (Illumina).

#### Sequence analysis

The QC and raw sequence pre-processing were performed using fastp (v. 0.20.0. April 2019) (11) with the following parameters: mean quality for 4 bp window size was 20, the adapter detection enabled, reads less than 120 bp were discarded, unmerged reads were included in the final FASTQ files. Total Sum Scaling (TSS) per-sample normalization was used to remove technical bias related to different sequencing depths among libraries and then scaled to units of reads per million per library. Taxonomic assignment was done using the naive Bayesian classifier method as implemented in the dada2 Bioconductor R package using an RDP training set (v.16).

### Statistical Analysis

All statistical analyses and graphing were performed using IBM SPSS V.23 (NY, United States) and GraphPad Prism V.6.0. (CA, United States), unless specified otherwise. Differences in demographic characteristics between groups were assessed using Independent-Samples Mann-Whitney *U* and Chi-Square Tests. An ELISA analyte was considered “detectable” if the measured analyte concentration was equal to or above the assay’s lowest level of detection (LLOD), and “undetectable” if the measured concentration was below the LLOD (Supplementary Table 1). Analytes were grouped into (i) “detectable in >50% of the participants” and analyzed as continuous variables using Independent-Samples Mann-Whitney U or (ii) “detectable in ≤50% of the participants” and analyzed as dichotomous variables. Differences among analyte concentrations across paired study visits were assessed by Friedman’s Two-way ANOVA by Ranks Test (across all three visits) or by the Related Samples Wilcoxon Signed Rank Test (between two visits). The α and β microbial diversities were estimated using Shannon’s diversity index and UniFrac weighted distance with Principal Coordinate Analysis (PCoA), respectively. Group comparisons for β-diversity were done via permutational multivariate ANOVA (PERMANOVA) of dissimilarity using adonis2 function from the vegan R package (12).

The linear discriminant analysis (LDA) effect size (LEfSe) algorithm was applied to identify features significantly different between the comparison groups with default settings (*p* ≤ 0.05 based on Kruskal-Wallis test and LDA score ≥2) (13). Statistical analysis of microbiome data was performed using MicrobiomeAnalystR package (14). Pearson correlation analysis between the OTU relative abundance, PASI scores, and IL1α were performed on Log10-transformed data. Data with missing values were excluded from the analyses.

## Results

### Participant Demographics and Clinical Characteristics

A summary of the socio-demographic characteristics and psoriasis diagnostic data are given in Table 1. Age, sex, or BMI did not significantly differ between groups. The median participant age was 33 years and significantly more individuals were not married in the psoriasis group (7/20) compared to the controls (1/20, *p* = 0.018). In the majority (85%) of the participants, one or both parents had psoriasis. Psoriasis vulgaris was most prevalent (75%) while 25% of the participants had guttate and palmoplantar psoriasis. Most participants (85%, 17/20) reported having had the condition for >5 years, indicating seasonality in symptom presentation. In most participants psoriasis was associated with hair, but not nail, damage. The median psoriasis area and severity index (PASI) score was 11.4; mild, moderate, and severe forms (15) were seen in 25, 35, and 40% of the participants, respectively (Table 1).

**TABLE 1:** Socio-demographic and psoriasis-specific characteristics of participants.

Participant characteristic	Psoriasis group (*N* = 20)	Controls (*N* = 20)	*P* value
Median age (IQR)	34.5 (31.0–37.8)	33.0 (31.3–34.0)	0.205
Men, *n* (%)	10 (50.0)	11 (55.0)	0.752
Mean BMI (range)	24.8 (21.4–28.7)	23.9 (18.6–32.7)	0.475
Married, *n* (%)	13 (65.0)	19 (95.0)	0.018
Psoriasis type			
Vulgaris	15 (75.0)	–	–
Guttate	3 (15.0)	–	–
Palmoplantar	2 (10.0)	–	–
Psoriasis present in a parent	17 (85.0)	–	–
Time since psoriasis first noted (years)			
<5	3 (15.0)	–	–
5–10	9 (45.0)	–	–
>10	8 (40.0)	–	–
Head hair damage present	17 (85.0)	–	–
Nail damage present	1 (5.0)	–	–
Median PASI (IQR)	11.4 (6.7–16.4)	–	–
Psoriasis severity based on PASI			
Mild [PASI < 7], *n* (%)	5 (25.0)	–	–
Moderate [7 ≤ PASI ≤ 12], *n* (%)	7 (35.0)	–	–
Severe [PASI > 12], *n* (%)	8 (40.0)	–	–

*BMI, body mass index; IQR, interquartile range; PASI, psoriasis area and severity index.*

### Comparison of Stool-Derived Cytokine Profiles

**FIGURE 1:**
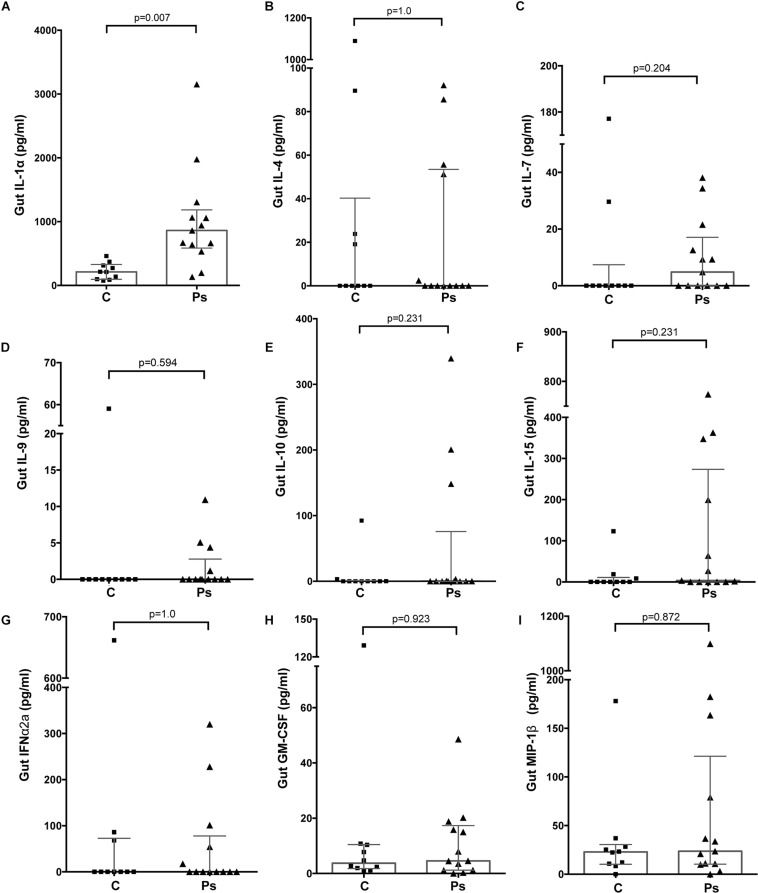
Differences in the levels of analytes representative of major functional groups of immune response mediators in the gut of individuals with (“Ps”) and without psoriasis (controls, “C”). **(A)** IL-1α (Proinflammatory); **(B)** IL-4 (Th1/Th2); **(C)** Il-7 (Homeostatic); **(D)** IL-9 (Th9); **(E)** IL-10 (Anti-inflammatory); **(F)** IL-15 (Growth factor); **(G)** IFN-α2 (Interferons); **(H)** GM-CSF (Colony-stimulating factor); **(I)** MIP-1β (Chemokine). For all depicted analytes data were available for 23/40 (psoriasis+, *N* = 13, and psoriasis-, *N* = 10) participants. Statistical significance assessed by the Independent-Samples Mann-Whitney *U* test. Source data are provided as a Supplementary Material.

**FIGURE 2:**
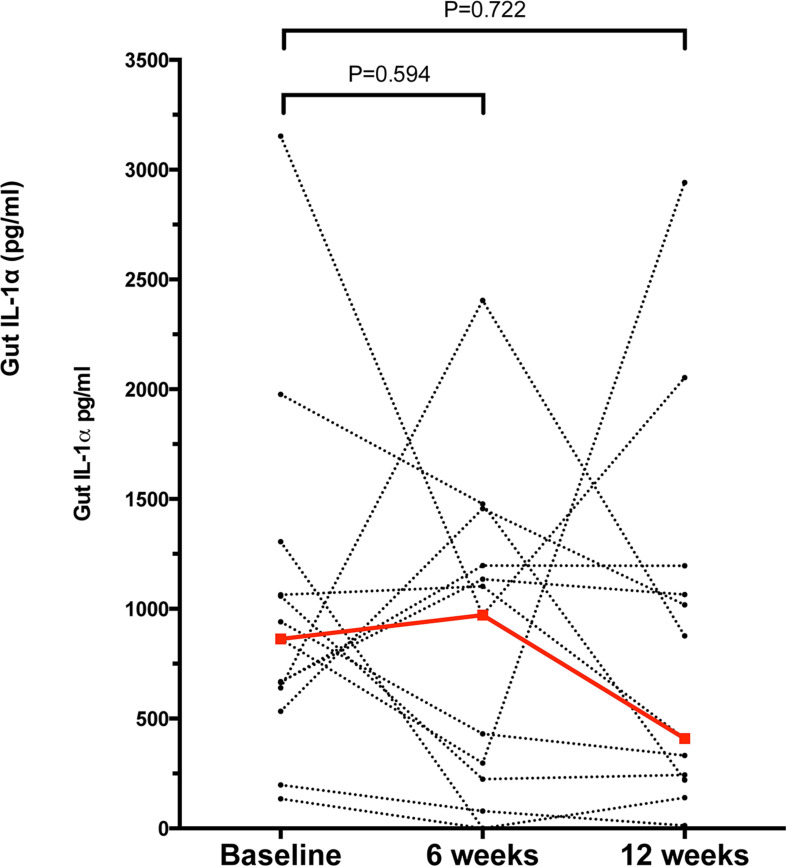
Concentration of gut IL-1α in the psoriasis group at baseline, 6 and 12 weeks after the initial study visit. Each dot represents a participant, dashed lines link samples paired across the study visits, *n* = 13 for paired comparisons. Red dots and line denote the median concentrations of IL-1α at each time point. Statistical significance assessed by the Related Samples Wilcoxon Signed Rank Test. Source data are provided as a Supplementary Material.

Samples from a total of 40 participants were analyzed. Cytokine and immunoglobulin data were obtained for 22 and 29 (out of a total of 40) participants, respectively. Repeated efforts to obtain ELISA data for outstanding measurements were unsuccessful, likely due to sample characteristics that were psoriasis-independent. Overall, 26/41 cytokines and chemokines and 3/6 Igs were detectable (i.e., >0 pg/ml in >50% of the participants); the psoriasis group tended to have higher median concentrations for most analytes (29/47 = 61.7%) compared to the control group (Supplementary Table 2); no correlation was observed between the stool analytes and the PASI scores. IL-1α was the only cytokine significantly elevated in the psoriasis group (4.19-fold, *p* = 0.007, Supplementary Table 2 and Figure 1A); this difference was not associated with differences in socio-demographic or psoriasis-specific characteristics in this subset of participants (*N* = 23, Supplementary Table 3). Notably, IL-1α levels remained significantly unaltered 6 and 12 weeks after the initial sampling (Figure 2), similar to the rest of the analytes, with the exception of IL-1β (*p* = 0.049) and IgG2 (*p* = 0.05), which fluctuated marginally over the period of 12 weeks after the first study visit (Supplementary Table 4).

### Comparison of Gut Microbiome Profiles

**FIGURE 3:**
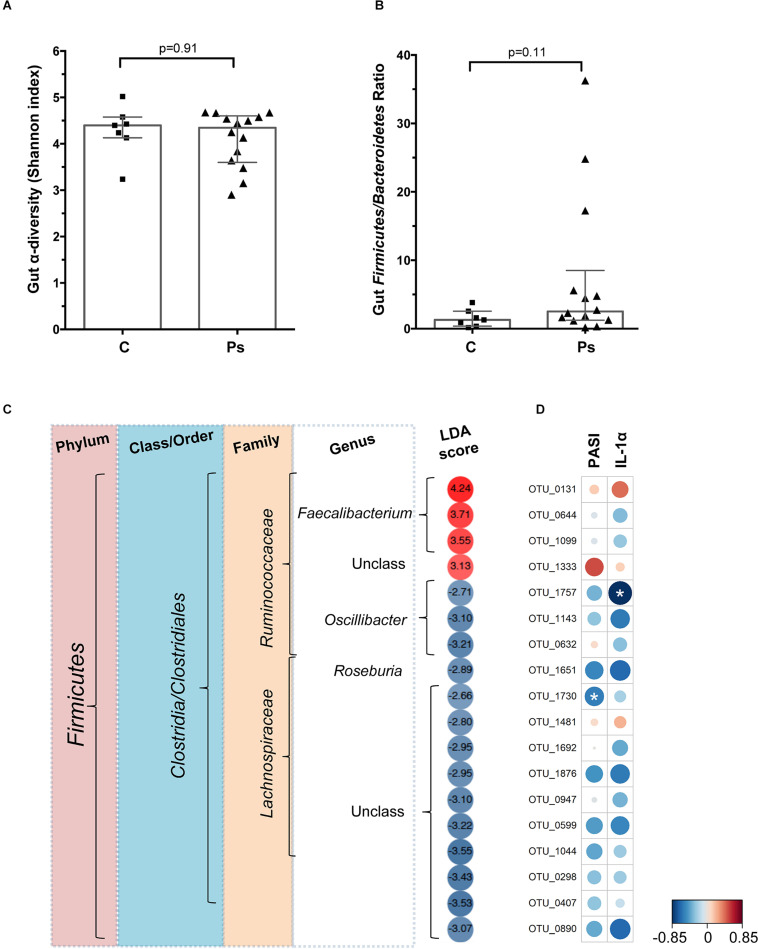
**(A)** Microbial species α-diversity measured by Shannon index in the gut of the psoriasis individuals and controls. **(B)** Gut *Firmicutes/Bacteroides* ratio of psoriasis + individuals and controls. **(C)** Heatmap showing the LefSe-derived LDA scores for each of the OTU (*n* = 18) differentially abundant in the gut of psoriasis + participants. Data based on *N* = 21 samples. **(D)** Heatmap based on the Pearson coefficients of correlation between the psoriasis-associated microbial taxa, baseline IL-1α and PASI scores. Analysis based on *N* = 14 samples. Significant correlations marked by asterisks (*).

*Gut microbiota diversity and Firmicutes/Bacteroides (F/B) ratio.* A total of 21 fecal samples, 14 psoriasis + and 7 controls, were available for the microbiome analysis. In our earlier analysis of the Kazakh gut metagenome, we found the *Firmicutes* to *Bacteroidetes* ratio to be significantly associated with metabolic syndrome. Therefore in the current analysis the *F/B* ratio comparison was a pre-specified endpoint. When compared to controls, neither the microbial diversity indices nor the *F/B* ratios of the psoriasis patients were significantly different (Figures 3A,B and Supplementary Figure 1), although the median *F/B* ratio in the psoriasis group was double of that observed in the controls (2.5 vs. 1.3), largely driven by an elevated F/B ratio in three psoriasis participants (Figure 3C).

*Bacterial taxa associated with psoriasis.* The LefSe analysis (12) identified 18 operational taxonomic units (OTUs) that could significantly discriminate among groups at *p* = 0.05 (Figure 3D). After correcting for multiple hypothesis testing, no OTUs remained significantly different between groups (FDR = 0.05); notwithstanding the overall differences in bacterial composition observed here are in agreement with those observed in other studies (5, 6). Specifically, all 18 OTUs that were found to be differentially abundant were from the phylum *Firmicutes* and, except one unclassified OTU, were classified as *Clostridia*, Order *Clostridiales*. The majority of these OTUs (14/18, 77.8%) were less abundant in psoriasis participants compared to controls. At the family level, all 8 OTUs from the Lachnospiraceae were found to be less abundant in psoriasis patients, while 4 of the 7 OTUs from the family *Ruminococcaceae* were more abundant in psoriasis patients and 3 were less abundant (Figure 3D). Examining this at the level of genus indicates that all OTUs from *Faecalibacterium* (*n* = 3 OTU) exhibited increased abundance, while those from the *Oscillibacter* (*n* = 3 OTU) and *Roseburia* (*n* = 1 OTU) exhibited reduced abundance in the psoriasis group (Figure 3D). Lastly, the correlation analyses confirmed that there was a negative correlation between OTU abundance and both disease severity (PASI) and IL-1α levels for most OTUs; only two of these correlations reached statistical significance (Figure 3D). Three taxa, OTU_0131 (from the genus *Faecalibacterium*), OTU_1333 (unclassified), and OTU_1481 (unclassified), exhibited positive correlations between OTU abundance and PASI and IL-1α (Figure 3D).

## Discussion

Here, we examined associations between psoriasis, and gut immunology and microbial parameters in an adult cohort based in Kazakhstan. We found that psoriasis was associated with elevated gut IL-1α and altered abundance of *Firmicutes*. Overall, these findings are consistent with the notion that psoriasis is linked to gut dysbiosis and inflammation (5, 6, 16, 17).

Few studies to date have directly assessed the immunological changes occurring in the intestine of psoriasis patients. Scher et al. reported elevation of soluble IgA and reduction of receptor activator of nuclear factor kappa-B ligand (RANKL), a critical factor controlling differentiation of intestinal lamina propria cells, in PsA (6). In the current study, we saw trends toward elevation for >60% of the analytes from the stools of psoriatic individuals, although only IL-1α, a cytokine central to the regulation of inflammation (18), was significantly elevated compared to the controls and remained unchanged in the psoriasis group for up to 3 months after the first visit to the clinic. Elevated cytokines in stool were previously detected in the context of gut inflammation (19, 20), while in a murine model of colitis IL-1α secreted by intestinal epithelium was the main driver of immune activation (21). In psoriasis, IL-1α drives the formation of dermal clusters of T cells and antigen presenting cells and is involved in the development of dermal Th17 responses (22–24). Therefore, it is possible that increased levels of gut IL-1α in individuals with psoriasis may contribute to increased inflammation via the gut-skin axis (25) and may help explain the well-documented epidemiological link between psoriasis and inflammatory bowel diseases (3).

To the best of our knowledge this study is the first to characterize psoriasis in a population from Kazakhstan and to assess its associations with gut immunology and microbiome. Earlier, we performed a large-scale metagenomic analysis and compared the Kazakh gut metagenome to its counterparts from other regions of the world (8). We found significant differences between the microbiomes of Kazakhs and both Europeans and East Asians. One distinguishing feature of the Kazakh gut metagenome is a remarkably dominant *Prevotella*-rich enterotype, which is typically regarded as proinflammatory (26), and was associated with the metabolic syndrome in our cohort (8). In our current analysis, psoriasis was not associated with changes in gut *Prevotella* abundance, but, consistent with studies from other cohorts, was associated with alterations in *Firmicutes* (5, 6). Although the direction of *Firmicute* change differs among studies, this suggests that psoriasis-associated gut microbiome signatures are generalizable to human populations residing in different parts of the world, but may be modified by differences in ethnic and environmental factors that are known to strongly influence the human gut microbiome composition (27).

We found that psoriasis was associated with an elevated abundance of members of the genus *Faecalibacterium*, which is consistent with a metagenomic analysis from Spain (5). On the other hand, a study based on a PCR-aided identification of specific bacterial species in the Netherlands found that stool samples from psoriasis patients were depleted for *Faecalibacterium prausnitzii* (4). Notably, gut *Faecalibacterium* alterations have been associated with eczema and IBD (28, 29) and, taken together, these data highlight the dynamic role gut *Faecalibacterium* spp. are potentially playing in the pathogenesis of both skin- and gut diseases.

The causality of the relationship between psoriasis and gut immunology and microbiota is unclear. Psoriasis is associated with systemic inflammation (1), which could drive changes in the gut mucosa and thereby affect the gut microbiota. It has also been proposed that the proinflammatory environment of the gut, precipitated by dietary insults and genetic predisposition, could induce systemic inflammation and thus cause skin-directed inflammation (30). Future mechanistic studies may clarify the exact mechanisms involved; meanwhile, our finding of enhanced IL-1α, a key regulator of inflammatory processes, suggests that the gut may play a central role in the induction of inflammatory responses in psoriasis (18). While current therapies for mild psoriasis target cutaneous manifestations and exhibit relatively low efficacies, the more efficacious therapeutic modes for severe psoriasis based on the use of antibodies are expensive and can have severe side effects (1). If the psoriatic disease is mediated by the gut immune milieu and microbiota, psoriasis treatment could benefit from modulating the gut microenvironment using microecologic agents, such as probiotics, and fecal microbiota transplantation.

Our findings should be interpreted in the light of several limitations. First, due to technical and logistic limitations our study had a small sample size and both the ELISA and metagenomic analysis results were only available for subsets of the original cohort. While the relatively small sample size is an important limitation of the study, this limitation was partially overcome by repeated cytokine measures, which confirmed, in particular, the consistently high levels of IL-1α in the psoriasis individuals compared to the controls. Notably, the significant fluctuation of gut IL-1β and IgG2 over the 3-month period may be due to a natural tendency of these signaling molecules to change over time or due to assay-related variation. Given the relatively small sample size of our metagenomic analysis, our analysis of psoriasis and gut microbiome interaction was likely under-powered and should be revisited in future, larger, studies. Further, soluble analytes were measured in stool supernatant, a highly heterogeneous sample, and further improvements of the protocol may improve cytokine yields; work is underway in our laboratory in this direction. Lastly, this study used an exploratory approach and therefore follow-up studies will be required to confirm these findings. It is noteworthy that despite age and sex matching, there were significant differences in the marital status of psoriasis participants and controls. Psoriasis is a socially stigmatizing condition that affects the individual’s ability to build relationships (1), underscoring the need for better therapeutic and psychological instruments to help individuals and their families affected by psoriasis.

In summary, this study for the first time assessed psoriasis associations with gut immunology and microbiome in an under-studied population from Central Asia. Although limited by a small sample size, the data presented here provide more evidence in support of the notion that gut immunology and microbiota may play critical roles in the pathogenesis of psoriasis, an autoimmune condition that is commonly thought to primarily affect the skin. Future studies are warranted to explore the inclusion of gut microenvironment modulating agents in the treatment and prophylaxis of psoriasis in this population.

## Data Availability Statement

All processed ELISA and microbiome data generated and analyzed during this study are included in the Supplementary Material. The raw microbiome sequence data are accessible through an online repository at: http://doi.org/10.5281/zenodo.3782581.

## Ethics Statement

All study procedures were approved by the University Medical Centre Ethics Committee. The patients/participants provided their written informed consent to participate in this study.

## Author Contributions

SY, DB, SG, BY, and AK: conceptualization and data curation. SY, DB, and SG: formal analysis and visualization. BY: funding acquisition. SK, LA, IK, AN, BY, and AK: methodology. BY, AK, and GH: project administration, resources, and supervision. SY and DB: writing – original draft. All authors: writing – review and editing.

## Conflict of Interest

DB was employed by Art Science LLP Innovative Center. The remaining authors declare that the research was conducted in the absence of any commercial or financial relationships that could be construed as a potential conflict of interest.
