# Supplementary material for: Psoriasis Is Associated With Elevated Gut IL-1α and Intestinal Microbiome Alterations
Source: Front Immunol. 2020 Oct 1;11:571319. doi: 10.3389/fimmu.2020.571319 (PMC7559734; doi:10.3389/fimmu.2020.571319)
Supplement: Supplementary file 1 [file Data_Sheet_1.PDF]

Psoriasis is associated with elevated gut IL-1α and intestinal microbiome alterations.

Sergey Yegorov[1,2]*, Dmitriy Babenko[3], Samat Kozhakhmetov [4], Lyudmila Akhmaltdinova[3], Irina Kadyrova[3], Ayaulym Nurgozhina[4], Madiyar Nurgaziyev[4], Sara V. Good[5], Gonzalo H. Hortelano,[1] Bakytgul Yermekbayeva[6], Almagul Kushugulova[4]

[1]School of Science and Humanities, Nazarbayev University, Astana, Kazakhstan

[2] Faculty of Education and Humanities, Suleyman Demirel University, Almaty, Kazakhstan

[3] Karaganda Medical University Research Centre, Karaganda, Kazakhstan

[4]Laboratory of Human Microbiome and Longevity, National Laboratory Astana, Nazarbayev University, Nur-Sultan, Kazakhstan

[5] Department of Biology, University of Winnipeg, Winnipeg, Canada

[6] University Medical Center Corporate Fund, Nur-Sultan, Kazakhstan

Supplementary Table 1. Description of the analytes assessed by multiplex ELISA in the stool supernatants of psoriasis patients and controls

#	Analyte	Functional category	Full name	Alternative name	Lowest limit of detection
1	IL-1α	Proinflammatory	Interleukin-1α	-	9.4 pg/mL
2	IL-1β		Interleukin 1β	-	0.8 pg/mL
3	IL-6		Interleukin 6	-	0.9 pg/mL
4	TNF α		Tumor necrosis factor α	DIF	0.7 pg/mL
5	TNF β		Tumor necrosis factor-β	Lymphotoxin-α (LT-α)	1.5 pg/mL
6	IL-17A		Interleukin 17a	CTLA8	0.7 pg/mL
7	sCD40L		CD40 ligand	IGM; IMD3; TRAP; CD154;	5.1 pg/mL
8	IL-2	Homeostatic	Interleukin 2	TCGF	1.0 pg/mL
9	IL-7		Interleukin 7	-	1.4 pg/mL
10	IL-12p40	Th1/Th2	Interleukin-12 p40	Interleukin-23	7.4 pg/mL
11	IL-12P70		Interleukin 12 p70	CLMF; NKSF; CLMF2; IMD28; IMD29; NKSF2;	0.6 pg/mL
12	IL-4		Interleukin 4	BSF1	4.5 pg/mL
13	IL-5		Interleukin 5	EDF; TRF	0.5 pg/mL
14	IL-13		Interleukin 13	P600	1.3 pg/mL
15	IL-9	Th9	Interleukin 9	P40; HP40	1.2 pg/mL
16	IL-1Ra	Anti-inflammatory	Interleukin-1 receptor antagonist	DIRA; IRAP; IL1F3; IL1RA; MVCD4;	8.3 pg/mL
17	IL-10		Interleukin 10	CSIF; TGIF; GVHDS	1.1 pg/mL
18	TGF-α		Transforming growth factor α	-	0.8 pg/mL
19	IFN-α2	Interferons	Interferon α2	-	2,9 pg/mL
20	IFN-γ		Interferon γ	IFG; IFI	0.8 pg/mL
21	IL-3	Growth factors	Interleukin 3	MCGF; MULTI-CSF	0.7 pg/mL
22	IL-15		Interleukin 15	-	1.2 pg/mL
23	EGF		Epidermal growth factor	URG; HOMG4	2,8 pg/mL
24	FGF-2		Fibroblast growth factor 2	BFGF; FGFB; HBGF-2	7,6 pg/mL
25	PDGF AA		Platelet-derived growth factor AA	PDGF1	0.4 pg/mL
26	PDGF AB/BB		Platelet-derived growth factor AB/BB	PDGF2	2.2 pg/mL
27	VEGF A		Vascular endothelial growth factor A	VPF; MVCD1	26.3 pg/mL

28	Fit-3L		Fms-related tyrosine kinase 3 ligand	CD135	5.4 pg/mL
29	G-CSF	Colony-stimulating factors	Granulocyte-colony stimulating factor	Colony-stimulating factor 3	1.8 pg/mL
30	GM-CSF		Granulocyte-macrophage colony-stimulating factor	Colony-stimulating factor 2	7.5 pg/mL
31	IL-8	Chemokines	Interleukin 8	CXCL8	0.4 pg/mL
32	Eotaxin		Eosinophil chemotactic protein	CCL11	4,0 pg/mL
33	Fractalkine		Fractalkine	CX3CL1, neurotactin	22.7 pg/mL
34	Gro		human growth-regulated oncogene	CXCL1	9.9 pg/mL
35	IP-10		Interferon gamma-induced protein 10	CXCL10	8.6 pg/mL
36	MCP-3		Monocyte-chemotactic protein 3	CCL7	3.8 pg/mL
37	MCP-1		Monocyte-chemotactic protein 1	CCL2	1.9 pg/mL
38	MDC		Macrophage-derived chemokine	CCL22	3.6 pg/mL
39	MIP-1α		Macrophage inflammatory protein 1-α	CCL3	2.9 pg/mL
40	MIP-1β		Macrophage inflammatory protein 1- β	CCL4	3.0 pg/mL
41	RANTES		Regulated on activation, normal t cell expressed and secreted	CCL5	1.2 pg/mL
42	IgA	Immunoglobulin	Immunoglobulin A	-	0.40 ng/mL
43	IgG1		Immunoglobulin G1	-	13.0 ng/mL
44	IgG2		Immunoglobulin G2	-	33.0 ng/mL
45	IgG3		Immunoglobulin G3	-	0.08 ng/mL
46	IgG4		Immunoglobulin G4	-	0.13 ng/mL
47	IgM		Immunoglobulin M	-	1.0 ng/mL

Supplementary Table 2. Analyte concentrations measured in stool supernatants of the psoriasis participants and controls.

Cytokine/Ig	Median concentration (IQR), pg/ml			Detectable in >50% of participants	P value*
	All participants	Controls	PSRS+		
**Cytokines and chemokines**					
EGF	0 (0-22.77)	0 (0-30.82)	0 (0-26.04)	yes	1
Eotaxin	2.92 (2.84-3.18)	2.89 (2.82-3.22)	2.98 (2.85-3.25)	yes	0.821
FGF-2	3.28 (0.99-6.97)	2.49 (0.89-28.71)	3.51 (1.23-5.79)	yes	0.974
G-CSF	0.92 (0.56-3.22)	0.74 (0.44-8.79)	1.69 (0.77-5.88)	yes	0.456
GM-CSF	4.55 (1.21-12.04)	3.73 (1.78-10.47)	4.58 (1.22-15.87)	yes	0.923
IL-1α	532.72 (210.66-1058.24)	215.34 (138.05-313.8)	862.37 (639.87-1064.22)	yes	0.007
IL-12p70	1.61 (0.54-14.11)	1.14 (0.07-3.81)	3.06 (1.61-56.97)	yes	0.08
IL-13	2.49 (2.22-2.88)	2.42 (2.16-3.27)	2.61 (2.3-2.85)	yes	0.722
IL-17A	1 (0-6.03)	0.46 (0-2.92)	1.57 (0-14.52)	yes	0.418
IL-1RA	5.95 (1.19-99.02)	2.26 (1.04-205.82)	17.12 (1.32-86.21)	yes	0.418
IL-3	2.51 (1.65-6.03)	2.74 (1.5-3.81)	2.42 (2.03-14.97)	yes	0.674
IL-5	0.9 (0.79-2.14)	0.92 (0.7-1.37)	0.9 (0.83-10.22)	yes	0.497
IL-8	2.71 (2.65-2.93)	2.69 (2.65-2.93)	2.76 (2.67-2.9)	yes	0.582
IP-10	26.86 (18.63-36.3)	26.86 (14.93-35.23)	31.43 (23.9-35.57)	yes	0.539
MCP-1	2.87 (2.71-4.11)	2.85 (2.7-3.21)	3.27 (2.78-34.75)	yes	0.381
MDC	1.5 (0-5.03)	1.65 (0-6.09)	1.66 (0-5.03)	yes	0.821
MIP-1 α	3.1 (3.09-3.23)	3.1 (3.08-3.16)	3.16 (3.1-3.25)	yes	0.203

MIP-1 β	22.86 (10.53-36.76)	22.86 (10.18-30.45)	23.63 (10.76-79.08)	yes	0.872
PDGF AA	1.91 (1.86-2.02)	1.9 (1.85-2.09)	1.94 (1.89-2.02)	yes	0.821
PDGF AB/BB	0.11 (0.08-4.41)	0.09 (0.08-0.23)	0.17 (0.08-646.12)	yes	0.418
RANTES	0.16 (0.16-0.17)	0.16 (0.16-0.18)	0.16 (0.16-0.17)	yes	0.582
sCD40L	2.44 (2.13-5.78)	2.43 (2.25-4.52)	2.66 (2.13-14.68)	yes	0.674
TGF-α	2.78 (2.73-2.83)	2.78 (2.74-2.87)	2.77 (2.73-2.78)	yes	0.582
TNF-α	2.42 (2.32-3.3)	2.35 (2.3-2.75)	2.5 (2.38-5.15)	yes	0.254
TNF-β	1.47 (1.43-1.58)	1.45 (1.43-1.66)	1.49 (1.43-1.54)	yes	1
VEGF-A	0.79 (0.39-2.83)	0.72 (0.45-8.36)	0.93 (0.38-4.57)	yes	0.771
FIt-3L	0 (0-0)	0 (0-1.51)	0 (0-0)	<50	0.571
Fractalkine	0 (0-27.85)	0 (0-51.38)	0 (0-107.68)	<50	0.646
GRO	0 (0-0)	0 (0-0)	0 (0-0)	<50	1
IFN-α2a	0 (0-57.76)	0 (0-72.62)	0 (0-54.3)	<50	1
IFN-γ	0 (0-1.33)	0 (0-2.31)	0.12 (0-1.79)	<50	0.415
IL-1β	0.86 (0-20.78)	0.11 (0-25.88)	9.35 (0-17.5)	<50	1
IL-10	0 (0-1.34)	0 (0-0.98)	0.22 (0-3.26)	<50	0.231
IL-12p40	0 (0-3.79)	0 (0-3.79)	0 (0-54.98)	<50	1
IL-15	0 (0-36.44)	0 (0-11.02)	3.5 (0-199.59)	<50	0.231
IL-2	0 (0-5.61)	0 (0-1.25)	0.23 (0-108.37)	<50	0.415
IL-4	0 (0-30.69)	0 (0-40.27)	0 (0-51.28)	<50	1
IL-6	0 (0-5.23)	0 (0-0)	0 (0-82.94)	<50	0.323
IL-7	0 (0-10.14)	0 (0-7.41)	4.82 (0-12.59)	<50	0.204

IL-9	0 (0-0)	0 (0-0)	0 (0-1.18)	<50	0.594
MCP-3	0 (0-8.6)	0 (0-0)	0 (0-119.65)	<50	0.323
**Immunoglobulins**					
IgA	3.28 (0.7-25.49)	2.13 (0.87-13.05)	7.33 (0.77-48.02)	yes	0.403
IgG1	4.1 (0-16.09)	0 (0-14.95)	6.8 (1.63-17.22)	yes	0.308
IgM	0.66 (0.46-0.95)	0.52 (0.45-0.7)	0.78 (0.5-1.01)	yes	0.211
IgG2	0 (0-0)	0 (0-0)	0 (0-0)	<50	1
IgG3	0 (0-0)	0 (0-0)	0 (0-0)	<50	1
IgG4	0 (0-0.06)	0 (0-0.02)	0.02 (0-0.14)	<50	0.433

Sample sizes: all (N=22-29), controls (N=7-10), psoriasis+ (N=12-19). *Independent-Samples Mann-Whitney U Test was used for analytes detectable in >50% of the participants, Chi-square tests were used for analytes detectable in <50% of the participants.

Supplementary Table 3. Socio-demographic and psoriasis-specific characteristics of participants with measurable IL-1α, N=23.

**Participant characteristic**	**Psoriasis group (N=13)**	**Controls (N=10)**	**P value**
**Median age (IQR)**	35.0 (31.0-37.5)	32.5 (31.0-35.3)	0.410
**Men, n (%)**	7 (53.8)	5 (50.0)	1.0
**Mean body mass index (range)^**	23.7 (18.6-29.0)	24.1 (18.6-32.7)	1.0
**Married, n (%)**	9 (69.2)	10 (100)	0.104
**Psoriasis type**			
Vulgaris	10 (76.9)	-	-
Guttate	2 (15.4)	-	-
Palmoplantar	1 (7.7)	-	-
**Psoriasis present in a parent**	5 (38.5)	-	-
**Time since psoriasis first noted**			
<5 years	2 (15.4.7)	-	-
5-10 years	5 (38.5)	-	-
>10 years	6 (46.2.0)	-	-
**Head hair damage present**	11 (84.6)	-	-
**Nail damage present**	1 (7.7)	-	-
**Median PASI (IQR)**	10.2 (6.0-18.0)	-	-

BMI, body mass index; PASI, Psoriasis Area and Severity Index

Supplementary Table 4. Analyte concentrations measured in stool supernatants of the psoriasis patients at 6 and 12 weeks after the initial study visit.

Cytokine/Ig	Median concentration (IQR), pg/ml			P value *
	Baseline	6 weeks	12 weeks	
EGF	0 (0-26.04)	0 (0-14.07)	0 (0-40.34)	0.819
Eotaxin	2.98 (2.85-3.25)	2.89 (2.82-3)	2.96 (2.82-3.05)	0.584
FGF-2	3.51 (1.23-5.79)	2.22 (1.17-6.06)	6.2 (1.05-10.02)	0.662
G-CSF	1.69 (0.77-5.88)	0.97 (0.51-2.01)	1.6 (1-31.17)	0.133
GM-CSF	4.58 (1.22-15.87)	2.97 (1.41-5.39)	12.04 (0.75-23.21)	0.269
IL-1α	862.37 (639.87-1064.22)	971.57 (224.47-1197.24)	409.96 (219.86-1065.08)	0.43
IL-12p70	3.06 (1.61-56.97)	3.82 (1.3-6.55)	5.21 (0.93-94.71)	0.943
IL-13	2.61 (2.3-2.85)	2.4 (2.2-3.09)	2.47 (2.14-3.2)	0.775
IL-17A	1.57 (0-14.52)	1.53 (1.02-4)	1.99 (0-9.92)	0.749
IL-1RA	17.12 (1.32-86.21)	1.28 (1.25-4.99)	2.14 (1.48-119.65)	0.092
IL-3	2.42 (2.03-14.97)	3.17 (2.47-3.35)	3.63 (1.9-31.82)	0.662
IL-5	0.9 (0.83-10.22)	0.97 (0.82-1.36)	2.25 (0.81-7.81)	0.735
IL-8	2.76 (2.67-2.9)	2.69 (2.64-2.71)	2.7 (2.65-2.75)	0.193
IP-10	31.43 (23.9-35.57)	30.29 (22.78-39.55)	28.3 (20.79-47.32)	0.584
MCP-1	3.27 (2.78-34.75)	2.8 (2.72-3.13)	3.23 (2.78-88.77)	0.589
MDC	1.66 (0-5.03)	0 (0-2.89)	0 (0-5.03)	0.552
MIP-1 α	3.16 (3.1-3.25)	3.11 (3.09-3.14)	3.12 (3.08-4.78)	0.47
MIP-1 β	23.63 (10.76-79.08)	29.93 (13.25-41.57)	37.46 (15.47-62.25)	0.097
PDGF AA	1.94 (1.89-2.02)	1.91 (1.89-1.96)	1.96 (1.9-2.03)	0.43
PDGF AB/BB	0.17 (0.08-646.12)	0.14 (0.1-1.22)	0.9 (0.11-306.1)	0.232
RANTES	0.16 (0.16-0.17)	0.16 (0.16-0.16)	0.16 (0.16-0.16)	0.449
sCD40L	2.66 (2.13-14.68)	2.73 (2.13-4.24)	3.75 (2.31-7.33)	0.5
TGF-α	2.77 (2.73-2.78)	2.76 (2.75-2.8)	2.81 (2.76-2.87)	0.092

TNF-α	2.5 (2.38-5.15)	2.41 (2.23-2.67)	2.39 (2.26-6.58)	0.397
TNF-β	1.49 (1.43-1.54)	1.47 (1.43-1.51)	1.5 (1.43-1.53)	0.673
VEGF-A	0.93 (0.38-4.57)	0.82 (0.46-26.3)	1.26 (0.47-162.73)	0.794
FIt-3L	0 (0-0)	0 (0-0)	0 (0-0)	0.646
Fractalkine	0 (0-107.68)	0 (0-0)	0 (0-263.93)	0.311
GRO	0 (0-0)	0 (0-0)	0 (0-29.36)	0.101
IFN-α2a	0 (0-54.3)	0 (0-0)	0 (0-110.12)	0.141
IFN-γ	0.12 (0-1.79)	0 (0-1.22)	0 (0-2.84)	0.54
IL-1β	9.35 (0-17.5)	0.95 (0-7.85)	2.95 (1.88-22.47)	**0.049**
IL-10	0.22 (0-3.26)	0.05 (0-0.49)	0.96 (0-50.33)	0.385
IL-12p40	0 (0-54.98)	0 (0-0.94)	0 (0-33.82)	0.629
IL-15	3.5 (0-199.59)	2.13 (0-13.4)	30.8 (0-109.51)	0.416
IL-2	0.23 (0-108.37)	0 (0-8.03)	0 (0-43.64)	0.819
IL-4	0 (0-51.28)	0 (0-0)	0 (0-31.98)	0.179
IL-6	0 (0-82.94)	0 (0-5.91)	6.26 (0-136.95)	0.185
IL-7	4.82 (0-12.59)	0.9 (0-7.33)	0 (0-7.57)	0.336
IL-9	0 (0-1.18)	0 (0-0)	0 (0-0)	0.846
MCP-3	0 (0-119.65)	0 (0-0)	0 (0-90.51)	0.772
**Immunoglobulins**				
IgA	7.33 (0.77-48.02)	11.57 (1.38-19.04)	4.17 (1.74-14.09)	0.538
IgG1	6.8 (1.63-17.22)	7.72 (0-15.16)	6.89 (1.45-15.03)	0.572
IgM	0.78 (0.5-1.01)	0.52 (0.4-0.86)	0.61 (0.53-0.78)	0.638
IgG2	0 (0-0)	0 (0-0)	0 (0-0)	**0.05**
IgG3	0 (0-0)	0 (0-0)	0 (0-0.04)	0.836
IgG4	0.02 (0-0.14)	0 (0-0.04)	0.02 (0-0.1)	0.29

***Difference between visits assessed by Friedman's Two-way ANOVA by Ranks Test.**

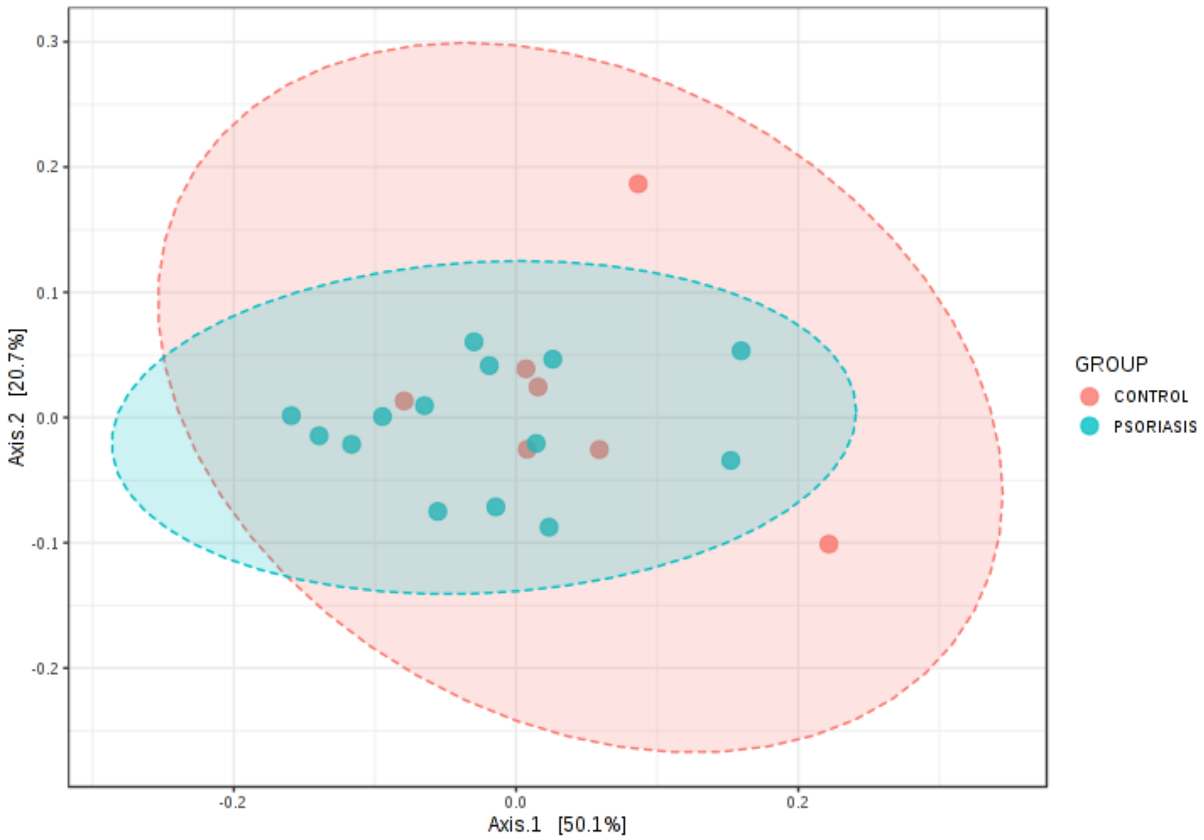

Supplementary Figure 1. Microbial species β-diversity measured in the gut of psoriasis+ individuals and controls
